# Characterization of the complete mitochondrial genome of *Eopsetta grigorjewi* (Herzenstein, 1890) (Chordata: Pleuronectidae) from the Yellow Sea and its phylogenetic implications

**DOI:** 10.1080/23802359.2022.2124826

**Published:** 2022-09-23

**Authors:** Dong Guo, Dandan Zhang, Zhong Tu, Long Yan, Weikuang Wang

**Affiliations:** a Liaoning Ocean and Fisheries Science Research Institute, Dalian, China; b Department of Environmental Engineering and Science, Feng Chia University, Taichung, Taiwan; c College of Life Science, Tarim University, Alaer, China; d Shandong Fisheries Development and Resource Conservation Center, Yantai, China

**Keywords:** Mitogenome, Pleuronectidae, *Eopsetta grigorjewi*

## Abstract

In this study, we determined the complete mitochondrial genome (mitogenome) of *Eopsetta grigorjewi* Herzenstein, 1890 (Chordata: Pleuronectidae) using Sanger sequencing technology. The total length of the mitogenome sequence of *E. grigorjewi* is 17,269 base pairs, including 13 protein-coding genes (PCGs), 22 transfer RNA genes, and two ribosomal RNA genes. The overall composition of the mitogenome is estimated to be 27.5% A, 25.6% T, 29.9% C, and 17.0% G. The phylogenetic relationships of 15 Pleuronectidae species were constructed based on the 13 PCGs by the maximum-likelihood approach using IQtree software.

The shotted halibut *Eopsetta grigorjewi* Herzenstein, 1890 (Chordata: Pleuronectidae) inhabits the northern parts of the Yellow Sea and Bohai Sea of China. The population of wild *E. grigorjewi* is not small, but studies of this species are rare. To obtain biological data for *E. grigorjewi*, we conducted germplasm analysis to determine its complete mitochondrial genome (GenBank accession no. MT768052). Our findings provide useful information for further studies of population genetics and phylogeny of *E. grigorjewi*.

We collected specimens of *E. grigorjewi* from the Dalian Sea area in Liaoning Province, China (39°12′32″ N, 124°45′38″ E). We extracted the total genomic DNA from muscle tissue using a modification of the phenol–chloroform procedure as described by Li et al. (2002). We amplified the complete mitogenome through long PCR, and purified products were sequenced using an ABI 3730 automatic sequencer (Applied Biosystems, Waltham, MA) at Liuhe Huada Biotechnology Company (Beijing, China) based on a primer-walking strategy. The gene annotation was performed using MITOS (Bernt et al. 2013) and ORFfinder, and the boundaries of protein-coding genes (PCGs) were further verified manually. The *E. grigorjewi* specimens used in this study are stored at the Key Laboratory of Mariculture & Stock Enhancement in North China’s Sea, Ministry of Agriculture and Rural Affairs, PR China, Dalian Ocean University, Dalian 116023, Liaoning, China (www.dlou.edu.cn, contact person: Professor Hao, email: haozhenlin@126.com) under voucher numbers DLOU-KLM-SSH236 to DLOU-KLM-SSH237.

The total length of the *E. grigorjewi* mitogenome is 17,269 base pairs, and the base composition is estimated to be 27.5% A, 25.6% T, 29.9% C, and 17.0% G. It contains 13 PCGs, two ribosomal RNA genes, and 22 transfer RNA genes. Most of the PCGs are encoded on the plus strand, but *nd6* is encoded on the minus strand. Most of the RNA genes are encoded on the plus strand, although nine transfer RNAs (*trnM*, *trnA*, *trnE*, *trnS*, *trnQ*, *trnC*, *trnP*, *trnN*, and *trnY*) are encoded on the minus strand. Among the 13 PCGs, 12 start with the conventional ATG codon and *cox1* starts with GTG. The most common stop codon is TAA, but some of the PCGs end with incomplete stop codons T or TA.

We used the 13 PCGs and a total of 15 species to resolve the phylogenetic position of *E. grigorjewi* using the maximum-likelihood method. Traditionally, Pleuronectiformes are divided into two suborders, Psettodoidei and Pleuronectoidei. Therefore, we chose *Psettodes erumei* from the suborder Psettodoidei to root the phylogenetic tree (Figure 1). *E. grigorjewi* grouped together with the clade formed by species from the families *Hippoglossus*, *Reinhardtius*, *Clidoderma*, and *Verasper*, and this grouping received the highest bootstrap support (BP = 100). However, the grouping of *Clidoderma* as a sister to *Verasper* received only weak support (BP = 48), possibly due to an insufficient phylogenetic signal in the dataset. In future studies, the use of a larger data matrix may resolve the phylogenetic relationships within Pleuronectoidei with higher resolution.

**Figure 1.**
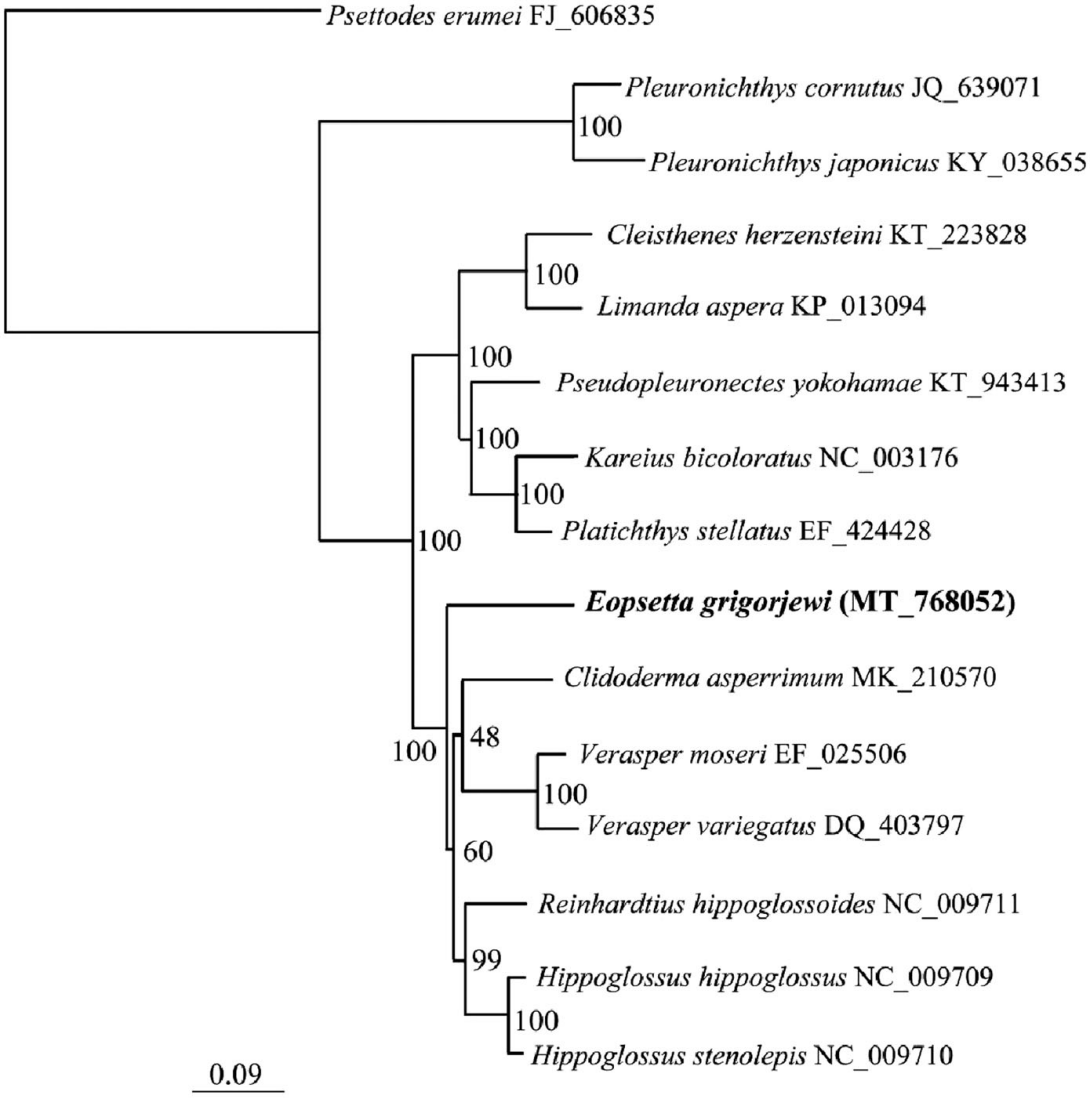
The phylogenetic position of *E. grigorjewi* was inferred using maximum-likelihood analysis based on 13 PCGs in IQ-TREE v1.6.1 (Nguyen et al. 2015). The maximum-likelihood searches were run using a combination of rapid hill-climbing approaches and the stochastic perturbation method with 1000 ultrafast bootstraps.

In addition, there is another sequence of *E. grigorjewi* in GenBank (OK545542) with second-generation sequencing technology, and this sequence is 348 nt shorter than the sequence in this study. Our study used the first-generation sequencing technology, and the extra part is the control region compared with *E. grigorjewi.* The control region of fish mitochondria mainly uses single nucleotide mutation and mutation sites for population differentiation, which are usually not used in phylogenetic analysis (Yang et al. 2008).

We expect that our results will contribute to molecular identification of this species and be helpful for exploring the phylogeny of Fish.

## Data Availability

The data that support the findings of this study are openly available in GenBank of NCBI at https://www.ncbi.nlm.nih.gov under accession number MT768052 or from the corresponding author.
